# Calcium status of multiparous Holstein cows during early lactation and its association with the health and production of 3 grazing dairies in Antioquia, Colombia

**DOI:** 10.3168/jdsc.2025-0945

**Published:** 2026-05-01

**Authors:** Geneva M. Graef, Aura M. Valencia, Valeria R. Cardona, Eduardo J. Bernal, Alejandra Z. Salazar, Alejandro C. Marquez, Thomas R. Overton, Francisco A. Leal Yepes

**Affiliations:** 1 Department of Animal Science, Cornell University, Ithaca, NY 14853; 2 Department of Agricultural Sciences, Universidad de Caldas, Manizales, Caldas, Colombia 170003; 3 School of Veterinary Medicine, University of Antioquia, Medellín, Antioquia, Colombia 050034; 4 Spin Colombia SAS, Bogotá, Colombia 110111; 5 Corpoleche, Belén, Medellín, Antioquia, Colombia 050030; 6 Department of Population Medicine and Diagnostic Sciences, Precision Livestock Health Laboratory, Cornell University, College of Veterinary Medicine, Ithaca, NY 14853

## Abstract

Periparturient hypocalcemia in dairy cows is known to negatively affect health and milk production. The grazing-based dairy industry of Colombia is the fourth largest milk producer in Latin America and is rapidly growing. However, with renewed commitments to sustainable intensification, Colombia may be disproportionately affected by poor forage quality and periparturient diseases due to limited access to technology and infrastructure. The primary objective was to assess the prevalence of subclinical hypocalcemia (SCH) across 3 commercial dairy farms in Antioquia, Colombia, and its association with milk production. Second, the study sought to evaluate associations of SCH with risk of disease and culling. Multiparous Holstein cows (n = 136) were enrolled in a prospective cohort study design on 3 farms in Antioquia, Colombia, from July to December of 2023. Serum concentrations of total calcium were analyzed prepartum, between 1 and 10 d before expected calving, as well as postpartum at 1, 2, 4, and 7 DIM. Cows were assigned either as eucalcemic (EU) or SCH based on the mean total calcium serum concentration at 4 DIM in this study (EU >2.40 mmol/L and SCH ≤2.40 mmol/L). Milk production was recorded daily. The SAS MIXED procedure with repeated measures was used to analyze differences between EU and SCH cows and respective milk production. The prevalence of SCH was 39%. Pre- and postpartum blood total calcium were greater in EU than SCH (differences prepartum = 0.23 mmol/L and postpartum = 0.37 mmol/L). Cows classified as EU had greater milk yield (difference 1.3 kg/d) and ECM (difference 1.0 kg/d) than SCH cows, without changes in risk to disease or culling. Overall, SCH is a relevant challenge in grazing systems in Colombian dairy herds and may affect the production and may unduly affect disease, culling, and reproduction outcomes.

## Highlights

• Subclinical hypocalcemia affects dairy cows, but prevalence in Colombia remains unknown.
• This study assessed SCH prevalence and production impacts in 136 grazing Holsteins.
• In this study, 39% of cows had SCH and lower serum calcium than eucalcemic cows.
• Study farms mirrored US dairies; eucalcemic cows produced more milk and energy-corrected milk versus SCH.
• Our results suggest SCH may affect overall herd performance in grazing dairy systems.

## Graphical Abstract

The geographic location of the 3 participating dairy farms in Antioquia, Colombia, and distribution of cows according to total blood calcium concentration at 4 days in milk (DIM) were analyzed. Cows were classified as eucalcemic (>2.40 mmol/L; 61%) or as having subclinical hypocalcemia (SCH; =2.40 mmol/L; 39%). Average milk yield differed between groups, with eucalcemic cows producing 34.0 kg/day compared with 32.6 kg/day for cows with SCH, indicating reduced milk production associated with lower calcium status during early lactation.

**Figure:**
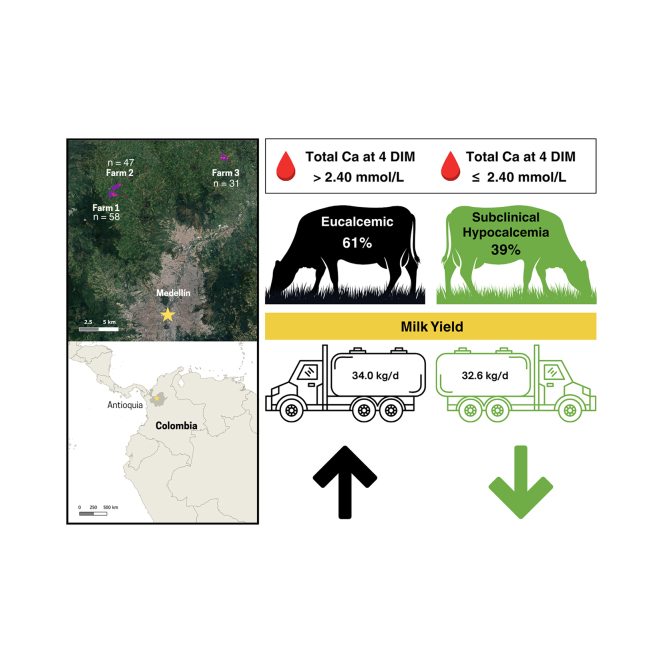

With a rising global population, the sustainable production of high-quality protein is essential (OECD-FAO, 2019). Livestock supplies 26% of global protein and occupies 80% of agricultural land, ~3,900 million hectares, most of which is for grazing ruminants (Herrero et al., 2015; OECD-FAO, 2019). South America contributes over 11% of global milk production, with over 3 million producers, and Colombia shows steady growth but faces challenges from variable forage quality and disease (FEDEGAN, 2019). The dairy industry in the south is heterogeneous, though the highlands of Antioquia boast increased size and specialization of dairy farms (Holmann et al., 2005). As grazing system size and efficiency rise, so do metabolic demands. Prolonged or delayed subclinical hypocalcemia (**SCH**) has been associated with reduced intake, health, fertility, and milk yield (**MY**) in confinement dairies (McArt and Neves, 2020; Seely and McArt, 2023) and temperate grazing systems (Roberts and McDougall, 2019). However, less is known about SCH risks and Ca dynamics in subtropical highland grazing systems, not only due to high variability in diagnostic thresholds of subclinical cases and timing of sampling (Sepúlveda-Varas et al., 2015; Melendez et al., 2023), but also the uniqueness of the high elevation, climate, and forage available (Martínez et al., 2018), the mix of small and medium specialized and dual-purpose systems (Durana et al., 2023), and the well-documented shifts in forage quality and mineral content of pastures (Roche and Berry, 2006). Given the known economic toll of SCH in confinement dairies, understanding it in grazing systems is vital for animal health and efficiency. The primary objective was to assess the prevalence of SCH across 3 commercial dairy farms in Antioquia, Colombia, and its association with milk production. Second, this study sought associations between SCH and disease risk and culling outcomes on these farms. Although specialized highland grazing dairies in Colombia are unique, they provide an excellent opportunity to assess SCH, given the additional risk of hypocalcemia faced by grazing cows. We hypothesized that cows in grazing systems would have an increased prevalence (≥50%) of SCH when compared with confinement systems, with similar negative effects on health and performance outcomes.

All procedures involving animals were approved by the Cornell University Institutional Animal Care and Use Committee (protocol #2023-0089). Three family-owned commercial dairy farms in Antioquia, Colombia, were enrolled between July and December 2023. Farms that previously participated in a study conducted by our group (Leal Yepes et al., 2020) were invited to participate in the current study and were enrolled if they met the following criteria: (1) the farm agreed to participate in the study, (2) the farm had >100 Holsteins, (3) the farm was primarily rotational grazing with some grain premix supplementation, (4) supplements did not include a Ca binder or anionic salt, and (5) the farm collected individual daily milk production data and milk quality data at regular intervals. Relevant information on farm size, location, and management protocols was collected at farm enrollment.

Health and production records were collected from farm records and Dairy Plan C21 (GEA Group Aktiengesellschaft, Germany). The 3 enrolled farms, on average, had 168.7 (±37.1; SD) lactating animals entering lactation 4.2 (±1.8; SD), and with reported average previous 305-d mature equivalent milk production (**ME305**) of 15,543 kg (±12,569 kg; SD). In a prospective cohort study design, Holstein cows were enrolled (n = 136) in this study when entering their second or greater lactation (parity 2, n = 57; parity ≥3, n = 79). When they were between 21 and 26 d before expected the calving date, they were moved to the farm's established prepartum paddock. Reported SCH prevalence in grazing systems can vary with the timing of the diagnostic sample and the selected cutpoint, ranging from 55% to 87%, average 71% (Melendez et al., 2023). In this study, a sample size calculation (n = 131) was conducted a priori to detect prevalence within 10% at 95% confidence, accounting for 10% attrition (Kane, 2017; Melendez et al., 2023). Body condition was scored weekly on a scale of 1 to 5 (Edmundson et al., 1989) through 4 wk postpartum as cows exited the milking parlor. Farm personnel diagnosed and treated clinical diseases based on their farm protocols and documented by research personnel: clinical hypocalcemia = cold extremities, trembling, and paresis; metritis = watery, fetid brown uterine discharge with or without systemic signs; retained placenta = retention of fetal membranes for >24 h; mastitis = abnormal milk or an inflamed quarter. Cows on all farms were milked twice daily at approximately 0300 and 1400 h. Individual milk weights were measured at each milking and recorded using an automatic measuring system from calving until 9 wk postpartum. Monthly milk samples were collected from the same single milking, continuously for a representative sample, and stored in vials containing bronopol preservative before immediate transport to the Milk Quality and Safety Laboratory for analysis (Laboratorio de Calidad de Leche de la Universidad de Antioquia, Medellín, Antioquia, Colombia). Milk fat, protein, and total solids were analyzed by infrared spectroscopy (FT + 400, Milkoscan FT +, FOSS Analytical, Hillerød, Denmark).

Blood samples were collected from the coccygeal vessels on one day between 1 and 10 d before expected parturition and at 1, 2, 4, and 7 DIM using a 20-gauge Vacutainer (BD Vacutainer, Becton, Dickinson and Company, Franklin Lakes, NJ) needle into 6-mL evacuated tubes without anticoagulants. Pre- and postpartum blood samples were collected between 0600 and 1000 h after completing milking. After sample collection, blood samples were immediately placed on ice and transported (≤30 min) to the laboratory, where serum was harvested after centrifugation at 2,000 × *g* for 20 min at ambient temperature (20°C–25°C) and split into aliquots (1.5 mL). Serum was stored at −20°C for up to 6 wk until analysis. One serum aliquot was analyzed for total Ca (**tCa**) at the Laboratorio de Calidad de Leche y Epidemiología Veterinaria (Universidad de Caldas, Manizales, Colombia). Laboratory using the compact semi-automated analyzer (X Monza, Randox Industries Ltd., Crumlin, United Kingdom). The calculated inter- and intra-assay CV were 14.5% and 7.3%, respectively.

Grazing management was similar on all 3 farms, consisting of continuous strip rotational systems with electric fencing moved every 2 to 3 d. Each pasture was given 27 to 36 d of rest between rotations. The botanical diversity of the pasture was ~95% Kikuyu grass (*Cenchrus clandestinus*) and 5% white clover (*Trifolium repens*). In addition to continuous forage availability, pelleted pre and postpartum concentrates were delivered to cows in a raised trough of the pre and postpartum paddocks respectively. No other supplementation was provided to the cows on the 3 dairy farms enrolled during the study.

Forage samples were collected weekly from July to December 2023 for close-up and fresh cows on 2 farms and later composited at 2-wk intervals. Using a 0.25 × 0.25 m quadrat and plate scale (VisionTechShop, Hackensack, NJ), 15 forage samples were collected per paddock (Dubbs et al., 2003; Martin et al., 2005) and dried on the high setting (100% power for maximum heat) in a microwave oven to determine DM (Bucholtz, 2007). Pre and postpartum concentrates were delivered monthly (Servicios Grupo BIOS S.A.S., Envigado, Antioquia, Colombia) to each farm and a sample was collected at delivery by farm personnel for this study. Both forage and concentrate samples were shipped for wet chemistry analysis at DairyOne (Ithaca, NY). Samples were analyzed for CP, adjusted CP, ADF (ANKOM Technology Method 14 Filter Bag Technique, DELTA), TDN (Goering and Van Soest, 1970), NEL, NEM, NEG, and minerals (CEM Corporation and Dairy One Forage Laboratory, 2016).

There were 2 wk throughout this study where no grass samples were collected due to (1) a vesicular stomatitis outbreak in the area that limited time on farms and movement between farms; and (2) severe weather conditions causing long-term power outages that prevented proper drying of samples. Logistical constraints restricted sampling of one farm to July through October 2023. It should be noted that DMI was not determined in this study.

All statistical analyses were conducted using SAS software (v. 9.4, SAS Institute Inc.). Pre- and postpartum data were analyzed separately. The primary outcomes of interest were the prevalence of SCH across 3 commercial, pasture-based dairies in Colombia and its associations with milk production outcomes (milk yield and components), disease risk, and culling risk, expressed as risk ratios (**RR**). Descriptive statistics were generated using the FREQ and MEANS procedures in SAS. Before analysis of MY, milk components, and blood mineral concentrations, cows were categorized based on mean serum tCa concentrations measured at 4 DIM to categorize cows as either eucalcemic (**EU**) or SCH, and to avoid misdiagnosis of those cows with only transient depletions of blood tCa concentrations that recover independently without loss of production or poor health outcomes (McArt and Neves, 2020). This approach is consistent with previously published studies in North American confinement dairy systems (Seely et al., 2021; Hernandez and McArt, 2023). In the present study, the mean serum tCa concentration at 4 DIM was 2.40 mmol/L (SD 0.50 mmol/L). Cows with serum tCa concentrations >2.40 mmol/L were classified as EU, whereas cows with serum tCa concentrations ≤2.40 mmol/L were classified as SCH.

The association between SCH and the risk of disease or removal was assessed using a fixed-effects multivariable Poisson regression via GENMOD in SAS. All cows were considered to have an equivalent time at risk for a disease or removal event at enrollment. Disease was diagnosed by farm personnel using farm protocols for diagnosis of clinical hypocalcemia, metritis, retained placenta, and mastitis. Potential covariates, BCS, and calving-related problems were tested for univariable associations with disease or removal outcomes using Fisher's exact tests. Covariates with *P* < 0.20 were included in each Poisson model. The Ca group (EU or SCH), previous ME305, and parity (2 or ≥3) were included in both models due to biological relevance.

Cow measures (BCS, blood tCa, average week MY, and components), were analyzed using mixed models with PROC MIXED in SAS, with a REPEATED statement for time. Pre- and postpartum periods were analyzed separately, with prepartum limited to 10 d before calving. Fixed effects included Ca group (EU vs. SCH), farm (1, 2, or 3), parity (2 or ≥3), time, and 2- and 3-way interactions, and were tested and removed in a backward stepwise manner if *P* > 0.10, only remaining in the model if *P* ≤ 0.10. Main effects of Ca group, farm, parity, time, and the interaction of Ca group by time remained in all cow models irrespective of significance. Forage and concentrate outcomes were similarly assessed using PROC MIXED including fixed effects of farm, time, and their interaction irrespective of significance. Significance was declared at *P* ≤ 0.05, and Tukey–Kramer adjustment was applied for multiple comparisons. Results are reported as least squares means ± 95% CI. The figure was generated using Microsoft Excel 2018 (Microsoft Corporation).

This study evaluated the prevalence of SCH on 3 Colombian dairy farms, based on serum tCa at 4 DIM, and its associations with MY, health, and culling in a grazing system. In total, 136 cows were enrolled in the study across the 3 farms between June and November 2023 (farm 1, n = 58; farm 2, n = 47; farm 3, n = 31). Overall, 39% of cows were SCH (parity 2 = 23%, n = 31; parity ≥3 = 34%, n = 46), less than the estimated 50% SCH in all confinement herds (Reinhardt et al., 2011) but greater than the ~30% of SCH cows that experienced either delayed or persistent declines in tCa concentrations (McArt and Neves, 2020). This outcome is within 11% to 60% of the reported range in more similar pasture systems of Chile and Brazil (da Silva et al., 2019; Melendez et al., 2023). There is still substantial variation in SCH diagnosis timing and thresholds, as shown by studies from Chile, Brazil, and this one.

Descriptive statistics of the population and prevalence of clinical health disorders identified by farm personnel are reported in Table 1. Parity, previous ME305, and prepartum body condition did not differ between EU and SCH cows (*P* > 0.05). Though not considered statistically significant (*P* = 0.12), the 3,782 kg (EU = 14,084 kg; 95% CI = 1,788–16,154 kg vs. SCH = 17,866 kg; 95% CI = 1,805–20,154 kg) difference in previous lactation ME305 between EU and SCH cows is noteworthy. Rebounding or low postpartum blood Ca concentrations from varied management strategies during the transition are likely to explain, at least in part, the wide range of production across lactations. Similar studies have linked greater parity and ME305 with greater milk fever risk in grazing systems (Saborío-Montero et al., 2017). In this study, cows considered SCH reported no significant changes (*P* ≥ 0.13) in risk of disease (EU n = 35 vs. SCH n = 22; RR = 0.84) or culling (EU n = 3 vs. SCH n = 8; RR = 2.75) during the first 60 DIM. The sample size calculation used for this study was based on detecting the herd-level prevalence of SCH. Therefore, this study is underpowered to detect any differences in diseases or culling. More research and larger studies are needed to better understand the associations of previous ME305 with SCH and the RR to other diseases and culling events in grazing herds of the global South.

**Table 1:** Descriptive statistics of the proportion of enrolled animals total, by parity, previous lactation, and weekly BCS of cows enrolled from 3 farms in Antioquia, Colombia^1^

Variable^3^	Calcium group^2^		*P*-value
	EU	SCH	
Cow count, n (%)	83 (61)	53 (39)	—
Disease count, n	35	22	—
Cull count, n	3	8	—
Farm, n			
1	34	24	0.22
2	34	13	—
3	15	16	—
Entering parity, n			
2	41	16	0.09
3+	42	37	—
Previous ME305, kg	14,084 (1,788, 16,470)	17,866 (1,805, 20,154)	0.12
BCS	3.0 (2.75, 3.25)	3.0 (2.75, 3.25)	0.62
Prepartum^4^ tCa, mmol/L	2.64 (2.51, 2.77)	2.41 (2.29, 2.54)	0.005
Postpartum tCa, mmol/L	2.51 (2.44, 2.58)	2.15 (2.09, 2.22)	<0.001
RR disease^5^	Referent	0.86 (0.58, 1.29)	0.49
RR culling	Referent	2.75 (0.76, 9.91)	0.13

1 Least squares means and 95% CI for prepartum and postpartum (0 to 7 DIM) serum total Ca (tCa). Final Poisson regression model outcomes of the risk of one or more disease or removal events within 60 DIM. All results are reported by postpartum Ca group.

2 Calcium grouping was categorized based on serum Ca (eucalcemic [EU] = Ca >2.40 mmol/L and SCH = Ca ≤2.40 mmol/L at 4 DIM). Final model for blood tCa = Ca group + farm + parity + (Ca group × week) + ɛ, where ɛ is residual or random error.

3 ME305 = previous 305 d mature equivalent milk production; RR = relative risk. Values in parentheses for previous ME305, BCS, and RR are 95% CI.

4 Prepartum samples were taken between 1 and 10 d before expected calving (5.9 d; 95% CI 5.1–6.3).

5 Outcomes include the diagnosis of clinical disease by farm personnel: clinical hypocalcemia = cold extremities, trembling, and paresis; metritis = watery, fetid brown uterine discharge with or without systemic signs; retained placenta = retention of fetal membranes for >24 h; mastitis = abnormal milk or an inflamed quarter.

Pre- and postpartum blood tCa concentrations are also presented for SCH and EU cows in Table 1. Prepartum samples were taken between 1 and 10 d before expected calving (5.9 d; 95% CI 5.1–6.3). Prepartum concentrations of tCa were greater (*P* = 0.005) in EU cows compared with SCH cows (difference 0.23 mmol/L, 95% CI 0.39–0.07 mmol/L) but were not different by parity (*P* = 0.30). In other studies, prepartum tCa alterations in SCH cows are rarely reported; this study's results might be influenced by various factors. First, they may be indicative of the large differences in previous ME305 production observed between groups (Neves et al., 2017; Roberts and McDougall, 2019), variable success between approaches to dry cow management, or even point different Ca dynamics in pasture systems or altered absorptive capacity of Ca resulting in preemptive depletions of Ca stores before calving (Roche and Berry, 2006). As anticipated, over time, postpartum tCa concentrations were greater in EU cows than SCH cows (difference 0.37 mmol/L, 95% CI 0.28–0.44 mmol/L; *P* ≤ 0.001; Table 1). Similarly, cows entering their second lactation have greater serum concentrations of tCa when compared with greater lactation animals (difference 0.15 mmol/L, 95% CI 0.07–0.24 mmol/L; *P* ≤ 0.001).

Milk yield, ECM, and milk composition for wk 1 to 9 are presented in Table 2 and Figure 1. More milk was produced by EU cows than SCH cows over time (difference 1.3 kg/d, 95% CI 0.6–1.9 kg/d; *P* < 0.001), and cows of parity ≥3 compared with second-parity animals (difference 1.5 kg/d, 95% CI 0.8–2.2 kg/d; *P* < 0.001). This persisted with ECM in both Ca group (difference 1.0 kg/d, 95% CI 0.3–1.7; *P* = 0.01) and parity (difference 2.3 kg/d, 95% CI 1.6–3.1 kg/d; *P* < 0.001). Regardless of SCH grouping, milk fat proportion (*P* = 0.04) and yield (*P* = 0.03) were greater in cows in their third or greater lactation compared with those entering their second lactation (differences of proportion 0.25%, 95% CI 0.01–0.50% and yield 0.10 kg, 95% CI 0.01–0.19 kg). Milk urea nitrogen was also increased in EU cows when compared with SCH cows (difference 1.3 mmol/L, 95% CI 0.8–1.5; *P* = 0.02). Understanding that hypocalcemia can impair metabolic and DMI recovery postpartum, the observed higher MUN in EU cows may be indicative of their improved fermentation activity and increased DMI, not a poor nitrogen efficiency, further supporting the presence of SCH in this study (Goff et al., 2020; Zhao et al., 2025). Finally, total solids numerically increased cows of parity ≥3 compared with cows entering their second lactation (difference 0.08 kg, 95% CI 0.23–0.32 kg; *P* = 0.09). Milk components, including protein, proportion of total solids, lactose, and BHB, were not different by Ca group (*P* ≥ 0.28) or parity (*P* ≥ 0.14). These results align with similar studies by Seely et al. (2021) and Neves et al. (2018), where delayed or prolonged subclinical hypocalcemia reduced MY. Larger studies are required to verify the effects of SCH grouping on milk components and ECM in pasture-based systems in the South American dairy industry.

**Table 2:** Least squares means and 95% CI of milk yield (kg/d), ECM (kg/d), and milk components for wk 1 to 9 for cows by calcium (Ca) status group^1^

Variable	Calcium group		*P*-value				
	EU	SCH	SCH	Farm	Parity	Time	Farm × time
Milk yield, kg/d	33.9 (32.8, 35.0)	32.6 (31.4, 33.5)	<0.001	<0.001	<0.001	<0.001	<0.001
ECM, kg/d	33.7 (33.2, 34.2)	32.7 (31.2, 32.3)	0.01	<0.001	<0.001	0.16	0.76
Fat, %	3.5 (2.0, 5.0)	3.4 (1.9, 5.0)	0.91	<0.001	<0.05	<0.001	0.28
Fat, kg	1.13 (1.07, 1.19)	1.09 (1.03, 1.15)	0.39	0.002	0.02	0.002	0.35
Protein, %	3.1 (3.1, 3.2)	3.1 (3.1, 3.2)	0.88	0.40	0.59	<0.001	0.46
Protein, kg	1.03 (0.31, 1.72)	1.0 (0.79, 1.28)	0.67	<0.001	0.44	0.21	0.04
Total solids, %	12.0 (11.8, 12.2)	12.0 (11.8, 12.2)	0.76	0.07	0.24	<0.001	0.39
Total solids, kg	4.00 (3.78, 4.12)	3.92 (3.75, 4.09)	0.81	<0.001	0.09	0.89	0.11
Lactose, %	4.8 (4.5, 5.1)	4.7 (4.3, 5.3)	0.28	0.06	0.14	<0.001	0.99
Lactose, kg	0.002 (0.001, 0.002)	0.002 (0.002, 0.002)	0.99	<0.001	0.49	0.03	0.01
BHB, mmol/L	0.01 (0.004, 0.02)	0.02 (0.01, 0.03)	0.70	0.01	0.88	0.02	0.09
MUN, mmol/L	16.3 (15.5, 17.1)	15.0 (14.2, 15.8)	0.02	<0.001	0.50	0.004	<0.001

1 Ca grouping was categorized based on serum total Ca (eucalcemic [EU] = Ca >2.40 mmol/L and SCH = Ca ≤2.40 mmol/L at 4 DIM). Final model for milk yields and components is as follows: milk measures = previous 305 d mature equivalent milk + Ca group + parity + farm + week + (Ca group × week) + (farm × week) + ɛ, where ɛ is residual or random error.

**Figure 1:**
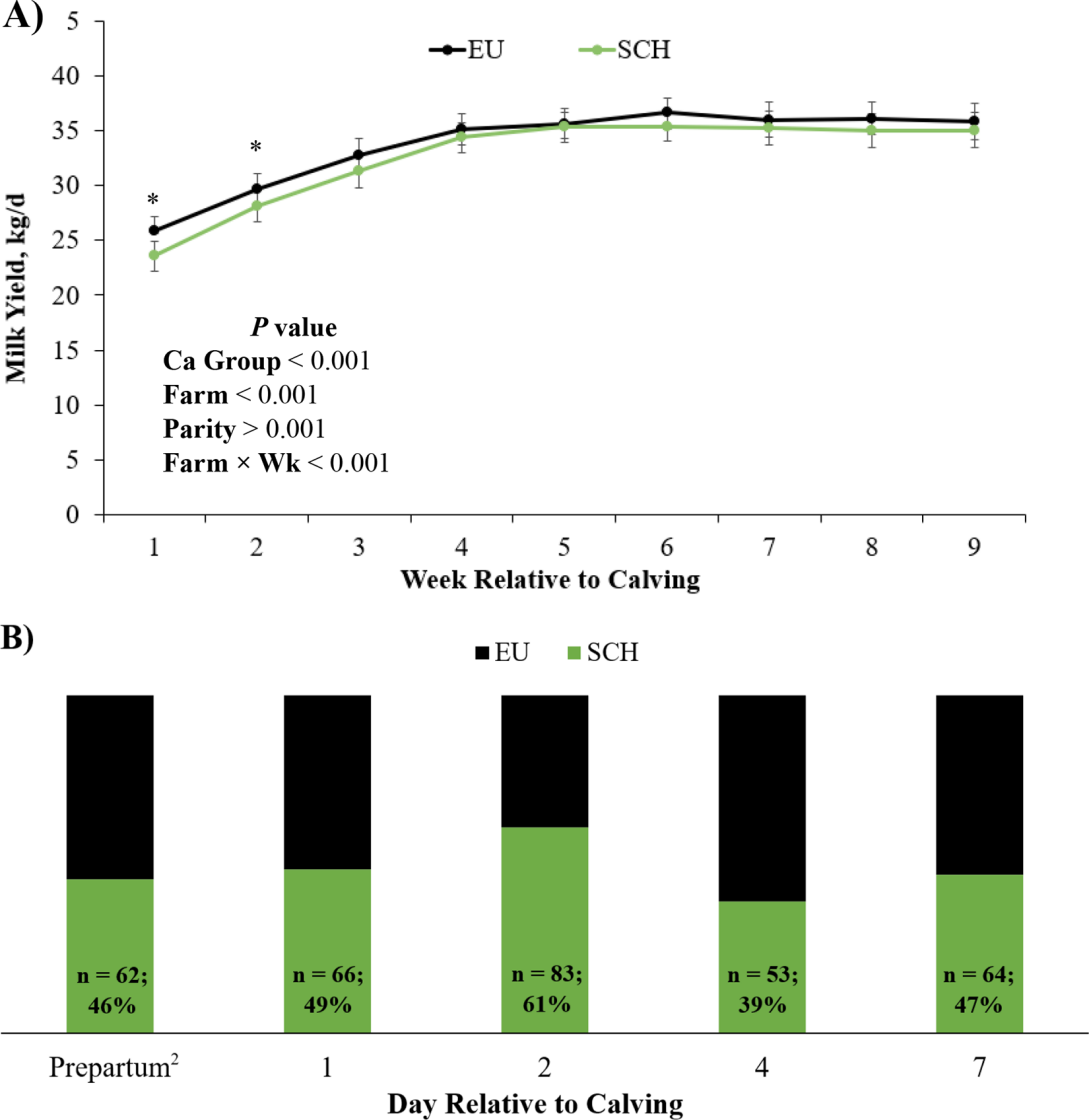
(A) Least squares means and 95% CI for milk yield from wk 1 to 9 postpartum for cows by calcium status group (subclinical hypocalcemia [SCH] = Ca ≤2.40 mmol/L; and eucalcemic [EU] = Ca >2.40 mmol/L at 4 DIM). *Indicates a difference in milk yield at *P* < 0.05. (B) Prevalence of SCH each day (SCH count, n; SCH % of total enrolled animals), diagnosed based on the mean total Ca concentration at 4 DIM. The prepartum samples were taken between 1 and 10 d before expected calving (5.9 d; 95% CI 5.1–6.3).

*Cenchrus clandestinum*, formerly known as *Pennisetum clandestinum*, is a C4 tropical grass brought to Colombia from Central and East Africa, for feeding animals at high altitudes including Antioquia (Mears, 1970; Correa et al., 2016). Wet chemistry results are reported in Supplemental Tables S1 and S2 (see Notes; Graef et al., 2026). Analyzed averages of DM in prepartum paddocks remained steady over the study period (18.4%; *P* = 0.15), though postpartum paddocks decreased linearly over time from 20.9% (95% CI 19.2–22.5%) to 16.6% (95% CI 15.0%–18.3%) over mo 1 to 5 (avg 17.8%, 95% CI 16.9%–18.6%; *P* ≤ 0.02). Small differences existed (*P* ≤ 0.05) in forage NFC, TDN, NEL, NEM, NEG, and iron (Fe) in the prepartum paddocks over time. The observed changes over time and seasonal differences from dry (July to August; December to March) to wet (September to November; April to May) seasons were indicative of the change in rainfall and temperature reported at higher elevations in Antioquia (Martínez et al., 2018; Lean and Golder, 2023). Forage NFC decreased over time (first month = 13.1% DM vs. fifth month = 9.5% DM; *P* = 0.02). Conversely, TDN, NEL, NEM, NEG, and Fe, increased linearly from mo 1 to 5 across all farms (*P* ≤ 0.05). Postpartum forage analysis over mo 1 to 5 also reported increases (*P* < 0.01) in TDN, NEM, NEG, phosphorus (P), and Fe. In terms of effects on cow Ca concentrations, these mineral differences are comparable to other works and not considered different enough to warrant disruption to Ca metabolism (Lean and Golder, 2023). Prepartum Ca, P, K, and DCAD analyses of forage and concentrate are relevant to Ca metabolism (Goff, 2018). The results of forage analysis in this study suggest that forage Ca (0.33%, 95% CI 0.25%–0.40%) and P (0.44, 95% CI 0.41%–0.47%) are unlikely to present a substantial risk of SCH (Goff and Koszewski, 2018). Potassium is a strong cation in the DCAD equation, and although elevated in this study (3.6%, 95% CI 3.5%–3.8%) and likely contributing to the high analyzed DCAD (402.3 mEq/kg, 95% CI 349.0–455.7 mEq/kg DM), it is considered normal for *C. clandestinum* and kikuyu grasses, particularly in the Colombian highlands (Lean and Golder, 2023). Overall grass production pre- and postpartum were consistent between farms (1,958 kg DM/ha, 95% CI 1,749–2,168 kg DM/ha; *P* = 0.48) but increased over time from dry to wet seasons (*P* < 0.001). Given similar rotation and fertilization among farms, similar production was expected. The variations were likely from seasonal changes in regional precipitation and temperature (Muñoz et al., 2026).

Interestingly, in this study, there was greater variation in the pre- and postpartum premixed concentrates fed to the cows in their respective groups. Analyzed premixed concentrate DM (87.1%; 95% CI 86.6%–87.5%), P (0.46%; 95% CI 0.41%–0.50%), Mg (0.24%; 95% CI 0.22%–0.26%), K (0.85%; 95% CI 0.76%–0.95%), Na (0.31%; 95% CI 0.24%–0.38%), and Cl (0.52%; 95% CI 0.43%–0.60%) decreased over mo 1 to 5 (*P* ≤ 0.05), whereas Fe increased from 213.7 mg/kg (95% CI 106.9–420.9 mg/kg) to 592.3 mg/kg (95% CI 200.9–791.5 mg/kg; *P* = 0.05). Commercial anion products and supplements can mitigate elevated prepartum DCAD caused by high K. However, the low anion content of the concentrate in this study, chloride (0.52%, 95% CI 0.43%–0.60%) and sulfur (0.26%, 95% CI 0.24%–0.28%), is inadequate to alleviate the elevated DCAD, and it is unlikely that the changes were large enough to meaningfully impact the cow's acid-base balance or Ca homeostasis (Goff, 2008). Postpartum concentrate composition similarly decreased over the same time frame (*P* ≤ 0.04) for DM, NEL, Ca. All concentrates were purchased from the same company, premixed before arriving at the farm as pellets to each farm before feeding.

Consistent sampling times and appropriate diagnostic cut points are both critical for identifying SCH, given global variation in feeding and management. Although 39% of enrolled cows in this study were diagnosed with SCH, the mixed results regarding disease RR, culling, and milk components highlight the need for larger studies to refine diagnostics. Conducted over 5 mo across similar farms in a single Colombian region, the study's limited duration limited seasonal analysis. Due to logistical constraints in shipping and forage analysis, samples were composited into 2-wk intervals, which may limit the study's ability to identify variability. Despite these constraints, findings link SCH with poorer performance and represent the first South American grazing study to classify cows using a time point at 4 DIM and tCa thresholds.

Overall, cows identified as SCH present a challenge in intensive grazing systems in Antioquia, Colombia. Over time, EU cows produced more milk; irrespective of lactation, though greater-parity cows (≥3 lactations), produced more milk fat. This study supports a growing body of work identifying the importance of SCH in grazing systems in the global South. Although further research is needed, it is evident that addressing SCH presents an opportunity to enhance grazing dairy systems in Colombia.
